# Comparison of Monoclonal Gammopathies Linked to Poliovirus or Coxsackievirus vs. Other Infectious Pathogens

**DOI:** 10.3390/cells10020438

**Published:** 2021-02-19

**Authors:** Jean Harb, Nicolas Mennesson, Cassandra Lepetit, Maeva Fourny, Margaux Louvois, Adrien Bosseboeuf, Sophie Allain-Maillet, Olivier Decaux, Caroline Moreau, Anne Tallet, Eric Piver, Philippe Moreau, Valéry Salle, Edith Bigot-Corbel, Sylvie Hermouet

**Affiliations:** 1 CRCINA, Inserm, Université de Nantes, Université d’Angers, 44000 Nantes, France; jean.harb@univ-nantes.fr (J.H.); nicolas.mennesson@univ-nantes.fr (N.M.); cassandra.lepetit@gmail.com (C.L.); maevafourny44@gmail.com (M.F.); margaux.louvois@orange.fr (M.L.); adrien.bosseboeuf@gmail.com (A.B.); sophie.allain@inserm.fr (S.A.-M.); edith.bigot@univ-nantes.fr (E.B.-C.); 2 Centre de Recherche en Transplantation et Immunologie UMR1064, Inserm, Université de Nantes, 44000 Nantes, France; 3 Laboratoire de Biochimie, CHU de Nantes, 44000 Nantes, France; 4 Internal Medicine, CHU de Rennes, 35000 Rennes, France; olivier.decaux@chu-rennes.fr; 5 Laboratoire de Biochimie, CHU de Rennes, 35000 Rennes, France; caroline.moreau@chu-rennes.fr; 6 Laboratoire de PGMC, CHU de Tours, 37000 Tours, France; anne.tallet@chu-tours.fr; 7 Laboratoire de Biochimie, CHU de Tours, 37000 Tours, France; piver_e@univ-tours.fr; 8 Inserm UMR1259, 37000 Tours, France; 9 Hematology Department, CHU Nantes, 44000 Nantes, France; philippe.moreau@chu-nantes.fr; 10 Internal Medicine, CHU Amiens, 80000 Amiens, France; Salle.Valery@chu-amiens.fr; 11 Laboratoire d’Hématologie, CHU Nantes, 44000 Nantes, France

**Keywords:** poliovirus, coxsackievirus, monoclonal gammopathy, MGUS, multiple myeloma, monoclonal immunoglobulin, infection, pathogen, antigen specificity

## Abstract

Chronic stimulation by infectious pathogens or self-antigen glucosylsphingosine (GlcSph) can lead to monoclonal gammopathy of undetermined significance (MGUS) and multiple myeloma (MM). Novel assays such as the multiplex infectious antigen microarray (MIAA) and GlcSph assays, permit identification of targets for >60% purified monoclonal immunoglobulins (Igs). Searching for additional targets, we selected 28 purified monoclonal Igs whose antigen was not represented on the MIAA and GlcSph assays; their specificity of recognition was then analyzed using microarrays consisting of 3760 B-cell epitopes from 196 pathogens. The peptide sequences PALTAVETG and PALTAAETG of the VP1 coat proteins of human poliovirus 1/3 and coxsackievirus B1/B3, respectively, were specifically recognized by 6/28 monoclonal Igs. Re-analysis of patient cohorts showed that purified monoclonal Igs from 10/155 MGUS/SM (6.5%) and 3/147 MM (2.0%) bound to the PALTAVETG or PALTAAETG epitopes. Altogether, PALTAV/AETG-initiated MGUS are not rare and few seem to evolve toward myeloma.

## 1. Introduction

Monoclonal gammopathies are characterized by the presence of a plasmacytic clone that produces large quantities of a single immunoglobulin (Ig), termed “monoclonal Ig.” Monoclonal gammopathy of undetermined significance (MGUS) is asymptomatic. However, over time MGUS acquire genetic alterations in clonal plasma cells and ~10% MGUS cases progress toward highly malignant multiple myeloma (MM) [1,2,3]. Recent studies of the antigens targeted by monoclonal Igs based on novel infectious antigen microarrays and immunoassays, indicated that chronic stimulation by an infectious pathogen or by a self-antigen, notably glucosylsphingosine (GlcSph), was a frequent pathogenic mechanism in MGUS and in MM [4,5,6,7,8,9,10]. Importantly, MM patients who presented with a GlcSph-reactive monoclonal Ig appeared to have a mild form of disease, while MM patients with an Epstein-Barr virus (EBV)-specific monoclonal Ig, who tended to present with severe forms of MM [6,7,8,9]. These advances are of great importance for therapy, since “target reducing” treatments that aim to suppress the target of the monoclonal Ig can be envisioned for MGUS patients, thus offering the possibility to prevent MM. For instance, eliglustat therapy aimed at reducing the level of GlcSph successfully reduced the amount of monoclonal Ig for two patients [11]. Target antigen reduction therapy could also improve the response of MM patients to classic chemotherapy, as observed with successful antiviral treatment for both MGUS and MM patients whose monoclonal Ig specifically targeted hepatitis C virus (HCV) proteins [12,13].

According to present knowledge, ~16% of MGUS and MM patients present with a monoclonal Ig specific for GlcSph, which suggests previously undetected chronic autoimmunity in these patients [9]. In addition, about half of MGUS or MM cases have a monoclonal Ig specific for an infectious pathogen, implying that a chronic, latent infection, initiated the monoclonal gammopathy in these patients [6,10]. In the above studies, the infectious pathogens identified as the targets of purified monoclonal Igs were mostly viruses, especially EBV, herpes simplex virus 1 (HSV-1) and HCV [6,9,10,14]. Four additional pathogens were reported to be the targets of monoclonal Igs: varicella zoster virus (VZV) in both MGUS and MM; cytomegalovirus (CMV) in MGUS and SM; and HSV-2 and *Helicobacter pylori* (*H. pylori*), in MGUS only [6]. Altogether, GlcSph immunoassays and multiplex infectious antigen microarrays can identify the target of a patient’s monoclonal Ig in ~60% IgG and IgA monoclonal gammopathies, a strong evidence for latent chronic diseases as initiating events. Hence, for ~40% cases, the target of the monoclonal Ig remains unknown. In the present study, we used commercial microarrays carrying 3760 epitopes from 196 pathogens, to analyze 28 MGUS purified monoclonal Igs of unknown specificity as assessed with the MIAA and GlcSph assays. These studies revealed that the peptide sequences PALTAVETG and PALTAAETG of the VP1 coat proteins of human polioviruses types 1 (PV1) and 3 (PV3) and coxsackieviruses B1 (CVB1) and B3 (CVB3), respectively, were novel targets of monoclonal Igs: these sequences were specifically recognized by 10 (6.5%) monoclonal Igs (all IgGs) from a cohort of 155 MGUS/SM and 3 (2.0%) monoclonal IgGs from a cohort of 147 MM. Monoclonal gammopathies linked to the PV and CVB PALTAVETG and PALTAAETG epitopes were then compared to monoclonal gammopathies associated with other infectious pathogens or with self-antigen GlcSph.

## 2. Materials and Methods

### 2.1. Patients

This study was promoted by the University Hospital of Nantes (#RC12 0085) with the approval of the local ethical committee and the Commission Nationale de l’Informatique et des Libertés (CNIL #912335).

We examined the serum of 302 patients (150 MGUS, 5 smoldering myeloma (SM), 147 MM). MGUS and SM patients, as well as 90 newly diagnosed MM (NDMM) were from the Centres Hospitaliers Universitaires (CHUs) of Tours, Rennes, Nantes and Amiens, in France, whereas 57 relapsed or refractory MM (RRMM) were from an international cohort provided by Novartis Pharma AG (Basel, Switzerland). Serum aliquots were collected and stored frozen over the 2010–2018 period. Written informed consents were obtained from all patients. The characteristics of the MGUS/SM and MM cohorts are described in Supplementary Table S1.

### 2.2. Separation of Monoclonal and Non-Clonal Igs

After clotting, blood samples were centrifuged at 2200× *g* (4 °C) and serum aliquots were frozen at −20 °C or −80 °C. Purification of monoclonal Igs and verification of their purity have been described previously [6,9,10,15]. After separation by electric charge on agarose gel electrophoresis (SAS-MX high resolution, Helena Biosciences, Gateshead, UK), bands corresponding to clonal Igs were carefully cut and proteins eluted from gels into phosphate buffer saline (PBS). Concentration of purified monoclonal Ig samples was determined using the Nanodrop Spectro-photometer ND-1000 with the IgG extinction coefficient (ε = 1.36 for a 1 mg/mL solution). Purity of each monoclonal Ig fraction was analyzed by isoelectrophoresis (isoelectrofocusing (IEF) using a range of pH 3–10) and immunoblotting onto polyvinylidene fluoride (PVDF) membranes. Membranes were then incubated with horse-radish peroxidase (HRP)-labelled anti-human IgGγ chain antibody for IgGs or anti-human IgAα chain antibody for IgAs (Dako, Glostrup, Denmark) [6,9,10,15]. This protocol has been validated by the verification of the purity of monoclonal Ig preparations using mass spectrometry [6]. 

### 2.3. Analysis of the Specificity of Recognition of Monoclonal Igs Using the GlcSph and MIAA Assays

The glucosylsphingosine (GlcSph) assay: As previously described, analysis of monoclonal Ig specificity for GlcSph was performed using an immunoblotting assay adapted from Nair et al. [7,9,10]. Briefly, PVDF membranes were incubated in 100 μg/mL of GlcSph in 0.1 M sodium bicarbonate, rinsed in PBS and 0.1% Tween 20 (PBST) detergent, then blocked with 5% bovine serum albumin (BSA) in PBST. Purified monoclonal Igs were submitted to agarose gel electrophoresis, then gels were blotted onto the GlcSph-saturated membranes by diffusion blotting [9,10,16,17]. After blocking with PBST and 2.5% BSA, membranes were incubated with peroxidase-conjugated AffiniPure donkey antihuman IgG (H+L) antibody (Jackson ImmunoResearch, West Grove, PA, USA) or HRP-conjugated goat antihuman IgAα chain antibody (Bethyl Laboratories, Montgomery, TX, USA), washed and revealed with Super Signal West Pico chemiluminescent substrate (Thermo Scientific).

The multiplex infectious antigen micro-array (MIAA) assay: The MIAA assay tests for proteins or lysates from EBV, HSV-1, HSV-2, CMV, VZV, HCV, H. pylori, Toxoplasma gondii (T. gondii), Borrelia burgdorferi (B. burgdorferi) [6,9,10,14,15]. Samples of serum and of purified monoclonal Igs were incubated on MIAA slides for 2 h at room temperature (RT). After washing, slides were incubated with a labelled secondary antibody (0.4 µg/mL Dylight^TM^ 680 Labelled Goat anti-human IgG (H+L), from Sera Care, Milford, MA, USA; Ref. 5230-0342 or DyLight^TM^680 goat anti-human IgAα chain from ImmunoReagent, Raleigh, NC, USA; Ref. GtxHu-001-E680NHSX). Fluorescence signal, detected with the Odyssey infrared imaging system scanner at a wavelength of 700 nm with a resolution of 21 μm (LI-COR, Lincoln, NE, USA) was quantified with the GenePix^®^ Pro 4 Microarray Acquisition & Analysis Software (Molecular Devices, Sunnyvale, CA, USA).

### 2.4. Analysis of the Specificity of Recognition of Monoclonal Igs Using PEPperCHIP^®^ Infectious Epitope Micro-Arrays

We used the commercial micro-arrays called PEPperCHIP^®^ Infectious Disease Epitope microarrays, from PEPperPRINT Gmbh (Heidelberg, Germany), which cover 3760 linear B-cell epitopes of 196 different pathogens associated with infectious diseases of the immune Epitope Database. The 3760 linear B-cell epitopes were translated into 4344 different peptides printed in duplicate (8688 peptide spots) and framed by poliovirus (KEVPALTAVETGAT, 42 spots), c-myc (EQKLISEEDL, 30 spots) and HA (YPYDVPDYAG, 40 spots) control peptides. Microarrays were placed in suited PEPperCHIP^®^ incubation trays (PEPperPRINT GmbH, Heidelberg, Germany). After 15 min pre-swelling in washing buffer and 30 min in blocking buffer MB-070 (Rockland, Gilbertsville, PA, USA), one PEPperCHIP^®^ Infectious Disease Epitope Microarray was incubated with the secondary goat anti-human IgG (Fc) DyLight680 antibody (1:5000) (Rockland, Gilbertsville, PA, USA) and control mouse monoclonal anti-HA (12CA5) DyLight800 antibody (1:2000) for 45 min at room temperature to analyze background interactions with the infectious disease epitopes. Subsequent incubation of PEPperCHIP^®^ Infectious Disease Epitope Microarrays with the purified monoclonal IgG (50 or 100 µg/mL) of patients was followed by staining with secondary and control antibodies for 30 min at room temperature and 200 rpm orbital shaking. The peptide microarrays were washed 3 × 1 min with PBST and rinsed with deionized water. After drying in a stream of air, images were recorded using an Odyssey Imaging System (LI-COR, Lincoln, NE, USA) at a wavelength of 700 and 800 nm with a resolution of 21 μm and a scanning sensitivity of for each channel 7. The HA control peptides were simultaneously stained as internal quality control to confirm the assay quality and the peptide microarray integrity. Image analysis and quantification of array data were done with PepSlide^®^ Analyzer (Sicasys Software GmbH, Heidelberg, Germany). These experiments were performed and results of the binding of purified monoclonal Igs were provided by the PEPperCHIP^®^ microarray manufacturer (PEPperPRINT).

### 2.5. Confirmation of the Recognition of Enterovirus VP1 Coat Protein Sequences by Monoclonal Igs Using Dot Blotting Assays with Peptides

Peptide Design and Synthesis. Six PV-derived peptides (2 relevant, 4 irrelevant) and three CVB-derived peptides (1 relevant, 2 irrelevant) were designed, then synthesized and purified at >95% (range: 95.2–97.8) by Covalab (Villeurbanne, France). The amino-acid sequences of the nine peptides are shown in Supplementary Table S2. All peptides were dissolved in DMSO, diluted in water, aliquoted and stored at −20 °C until use. 

The PV/CVB Dot Blotting Assay. Peptides (0.9 µg in 1 µL, 0.5 nmol) were spotted on an Amersham^TM^ Protran^TM^ 0.45 µm nitrocellulose membrane (GE healthcare Life Sciences, Chicago, IL, USA) and the membrane was let to dry. After incubation in PBS for 10 min, the membrane was saturated with PBST + 5% nonfat dry milk overnight at RT. Fifty µL of serum (0.4 g/L) or 10 μg of purified monoclonal Ig (0.2 g/L) in PBST + 1% BSA were added to the membrane and incubated for 2 h at RT. The membrane was washed with PBST, then incubated at RT for 1 h with peroxidase affinipure donkey anti-human IgG (H+L) (Jackson Immuno Research, West Grove, USA) diluted 1:10000 in PBST + 1% BSA for monoclonal IgGs or HRP-labelled goat anti-human IgA (α chain) from Bethyl Laboratories (Montgomery, TX, USA) for monoclonal IgAs, diluted 1:5000 in PBST + 1% BSA. After washes in PBST, membranes were incubated with Super Signal West Pico or Femto chemiluminescence kits (Thermo Scientific, Rockford, IL, USA) and immune complexes were revealed using Camera Azure BioSystems c500 Imager (Azure Biosystems, Dublin, CA, USA).

### 2.6. Statistics

Data analysis was performed by GraphPad Prism 6.01 software. Patient parameters were expressed as medians and ranges, or/and means ± standard error of the mean (SEM). The *Chi-2* test was used for categorical variables. The tests used are indicated in the legends of Figures and Tables. A *p* value below 0.05 was considered statistically significant.

## 3. Results

### 3.1. Characteristics of Patients and Monoclonal Igs

In this retrospective study, we analyzed serum samples collected from 302 patients with a monoclonal Ig (150 MGUS, 5 SM, 147 MM). For MGUS patients, the monoclonal Ig was an IgG for 137 cases and an IgA for 13 cases. The 5 SM patients all had a monoclonal IgG. For MM patients, the monoclonal Ig was an IgG for 125 cases and an IgA for 22 cases. Supplementary Table S1 shows the main characteristics of the cohorts of MGUS/SM and MM patients (90 NDMM and 57 RRMM). The sex ratio and median age of patients were similar for the MGUS/SM and NDMM cohorts but patients with RRMM were younger (61.0 year old) and more frequently female (70%). The median quantity of monoclonal Ig reflected disease progression, over a range from 16.0 g/L for MGUS/SM and 23.0 g/L for NDMM, to 34.8 g/L for RRMM. 

After separation from the non-clonal Igs in blood serum, the specificity of recognition of purified monoclonal Igs was analyzed using the GlcSph immunoblot and MIAA assays, as described for MGUS and NDMM patients [6,9,10]. A new cohort of 57 RRMM patients was thus analyzed. These studies allowed to identifying the target of 180 monoclonal Igs; among those, 14 were from RRMM patients (Supplementary Table S1, Supplementary Figure S1), 46 were from NDMM patients and 120 were from MGUS/SM patients [6,9,10]. The identified targets of the purified monoclonal Igs were as follows: GlcSph: n = 6 for RRMM patients (Supplementary Figure S1), n = 14 for NDMM patients and n = 25 for MGUS/SM patients; and MIAA infectious pathogens: n = 8 for RRMM patients (Supplementary Table S1) and n = 32 for NDMM patients and n = 95 for MGUS/SM patients, as published [6,9,10]. The percentage (%) of patients with a GlcSph-specific monoclonal Ig was not significantly different in MGUS/SM, NDMM and RRMM, respectively 16.1%, 15.5%, 10.6%. In contrast, the % of patients with a monoclonal Ig specific for an infectious pathogen of the MIAA was highest for MGUS/SM (61.3%) then sharply decreased for NDMM (35.6%, *p* = 0.0001 vs. MGUS/SM) and RRMM (14.0%, *p* < 0.00001 vs. MGUS/SM and *p* = 0.0043 vs. NDMM) (Supplementary Table S1). Hence the % of monoclonal Igs of unknown specificity was highest for RRMM patients (43/57 or 75.4%) compared to NDMM (44/90 or 48.9%, *p* = 0.0014) or both cohorts of MM compared to MGUS/SM (35/155 or 22.6%, *p* < 0.0001 vs. RRMM and *p* < 0.0001 vs. NDMM). 

Altogether, the monoclonal Ig of 122 patients (33 MGUS, 2 SM, 87 MM) lacked an identified target after these analyses. The infectious specificity of a fraction of these monoclonal Igs was then further analyzed using PEPperCHIP^®^ Infectious Epitope microarrays.

### 3.2. Epitope Recognition by Monoclonal Igs as Assessed by PEPperCHIP^®^ Infectious Epitope MicroArrays

**Figure 1:**
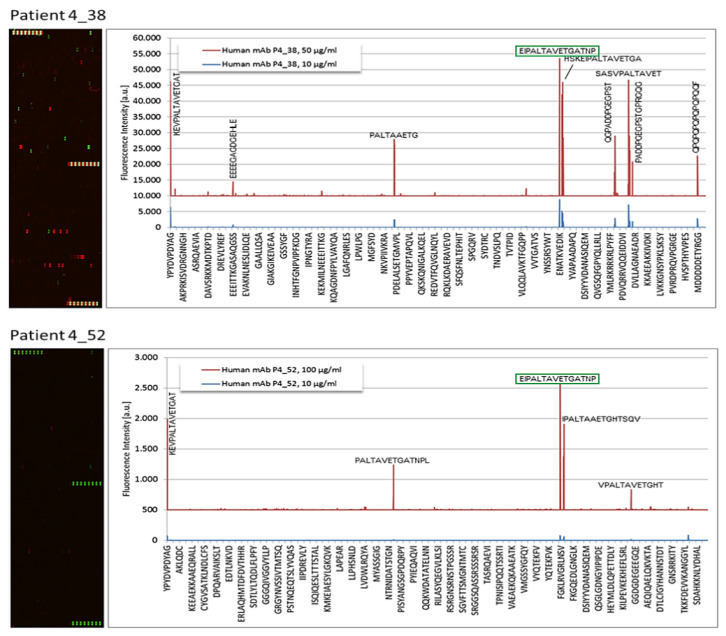
Results of PEPperCHIP*^®^* Infectious Epitope MicroArrays obtained for Monoclonal IgGs Specific for VP1 Coat Protein of Human Polioviruses.Microarrays were incubated with the patient monoclonal IgG (50 or 100 µg/mL), stained with secondary and control antibodies, then read out at scanning intensities of 7/7 (red/ green) (see Materials and Methods). Antibody response against peptides is annotated next to corresponding signals in the intensity plot (left panel). Well-defined staining of HA control peptides appear in green; PV control peptides appear in red.

**Figure 2:**
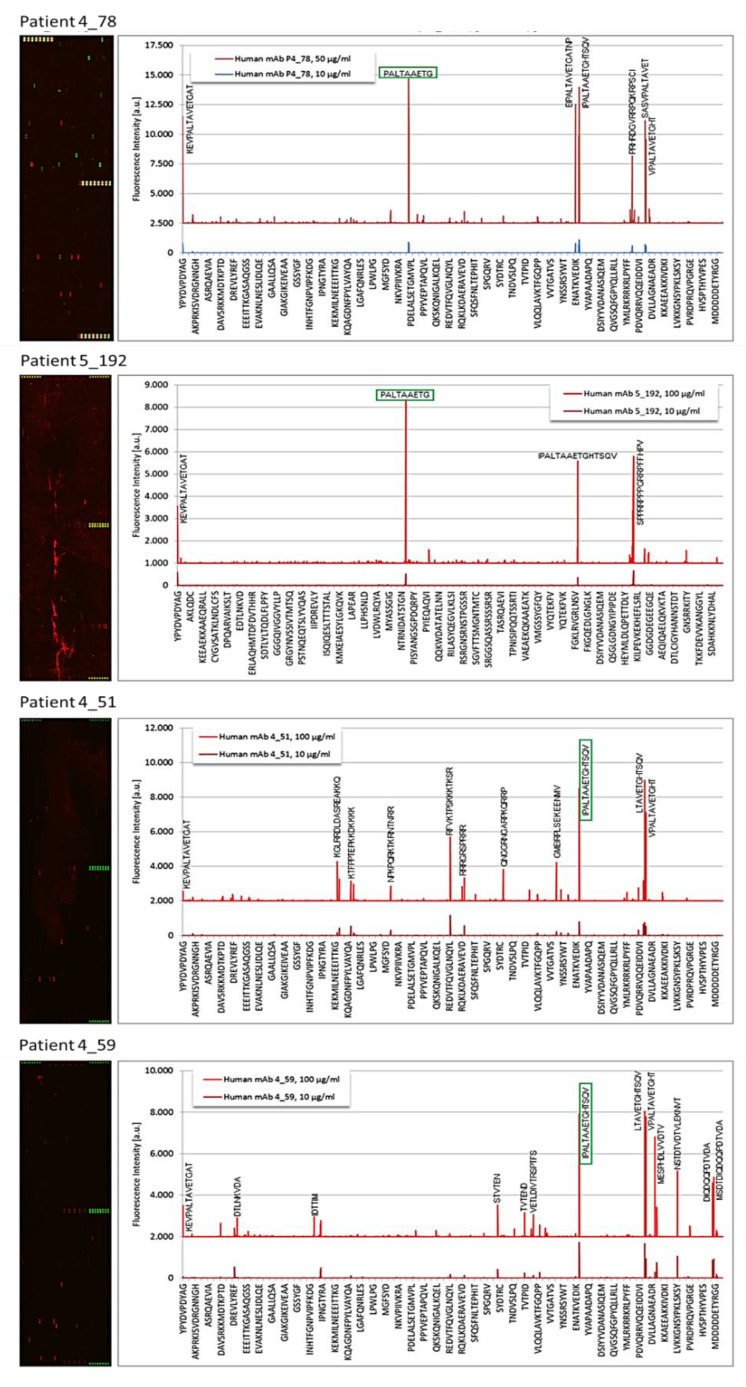
Results of PEPperCHIP*^®^* Infectious Epitope MicroArrays obtained for Monoclonal IgGs Specific for VP1 Coat Protein of Human Coxsackieviruses. Microarrays were incubated with the patient monoclonal IgG (50 or 100 µg/mL), stained with secondary and control antibodies, then read out at scanning intensities of 7/7 (red/green) (see Materials and Methods). Antibody response against peptides is annotated next to corresponding signals in intensity plots (left panels). Well-defined staining of HA control peptides appear in green, PV control peptides appear in red.

For reliable interpretation, PEPperCHIP^®^ arrays required 50–100 μg of purified monoclonal Ig. Because of insufficient volumes of serum for MM patients, PEPperCHIP^®^ arrays were performed for 27 MGUS patients and 1 SM patients with sufficient volume of serum; all 28 patients had a monoclonal IgG. An additional monoclonal IgG identified by the MIAA assay as targeting EBV EBNA-1 was used as positive control. The results obtained with the microarrays allowed interpretation for 24 of the 29 monoclonal Igs (82.8%). Fluorescence intensity (FI) > 1500 was considered significant (Figure 1 and Figure 2, Supplementary Figures S2 and S3). As expected, the EBNA-1-specific control monoclonal IgG bound specifically to the EBV EBNA-1 PPRRPPPGRRPFFHPVG sequence (FI: 3786) (Patient 4_07, Supplementary Figure S2). The PEPperCHIP^®^ arrays were negative for 11 MGUS patients: the monoclonal IgG did not bind to any peptide in the array for these patients (FI < 1500). For 2 MGUS, the monoclonal IgG bound to EBV EBNA-1 peptides that contained the PGRRPFF epitope (PPGRRPFFHPVGEADYF, patient 4_11; GRRPFFHPVGEADYFEY, patient 4_19) (Supplementary Figure S2). For 5 patients, the monoclonal IgG bound to envelope glycoprotein G from HSV-1 (3 MGUS, 1 SM) or HSV-2 (1 MGUS) but target amino-acid sequences differed: EGAGDGE (patients 2_91, 4_12), MPSIGLEEEE (patients 4_09, 4_89) and LPQSPGAFPLAE (patient 4_98) (Table 1, Supplementary Figure S3). 

For the remaining 6 MGUS patients, the microarrays revealed that the purified monoclonal IgG bound strongest to the peptide sequences PALTAVETG or/and PALTAAETG, found in the VP1 coat protein of human PV1 and PV3 (PALTAVETG, patients 4_38 and 4_52; see Figure 1 and Table 1) and CVB1 and CVB3 (PALTAAETG, patients 4_51, 4_59, 4_78, 5_192; see Figure 2 and Table 1). The FI observed for these monoclonal Igs ranged from 1657 to 43,579. The median FI was 6918 for PV/CVB VP1 coat protein, 11,351 for EBV EBNA-1 and 9243 for HSV-1/2 envelope glycoprotein G.

### 3.3. Confirmation of Poliovirus and Coxsackievirus VP1 Recognition by PALTA**V/A**ETG-Specific Monoclonal Igs Using Dot Blotting Assays

A dot blotting assay was set up that carried 9 peptides derived from the VP1 coat protein of PV and CVB, including the PALTAVETG and PALTAAETG epitopes identified with the PEPperCHIP^®^ arrays (Supplementary Table S2). The aim of this “PV/CVB assay” was two-fold: firstly, to confirm the recognition of VP1 coat protein PALTAV/AETG epitopes by monoclonal IgGs; secondly, to try to determine which virus (PV1 or PV3, CVB1 or CVB3, other?) was the target of monoclonal Igs. Therefore, the sequences of the peptides of the dot blotting assay were either identical to the amino-acid sequence of VP1 coat proteins of PV1 (“PV1” peptide THSKEIPALTAVETGATN), PV3 (“PV3” peptide AHSKEVPALTAVETGATN), CVB1 or CVB3 (common “CVB” peptide TNSESIPALTAAETGHTS). Six other peptides were irrelevant peptides, used as negative controls (Supplementary Table S2). We also used two controls from the MIAA assay. The positive control was Accurun^®^ 40 series 5000 Analytes (Sera Care, Grenoble, France), which includes antibodies to measles, VZV and other infectious pathogens, diluted in serum. The negative control was Viroclear^®^ToRCH (Biorad, Marnes la Coquette, France), based on serum confirmed to be negative for IgG and IgM to several pathogens, including measles virus, CMV, HSV-1, HSV-2 and T. gondii. The two controls were tested for the presence (positive control) or absence (negative control) of antibodies to the PALTAVETG and PALTAAETG sequences.

Membranes were spotted with the following peptides: (a) HP-1a, HP-3a and HC-a, respectively relevant to PV1, PV3 and CVB1/B3; (b) irrelevant HP-1b, HP-3b and HC-b peptides; (c) irrelevant HP-1c, HP-3c and HC-c peptides (see Supplementary Table S2), then incubated with positive or negative controls or serum from patients. After validation that no signal was obtained with irrelevant peptides for serum samples, purified monoclonal Igs were incubated with relevant peptides only. Immunoblot revelation with secondary antibodies (Materials & Methods). The assay confirmed that the purified monoclonal IgG of MGUS patients 4_38, 4_51 and 4_52 (in bold, left panel) bound specifically to PALTAVETG or PALTAAETG epitopes.

**Figure 3:**
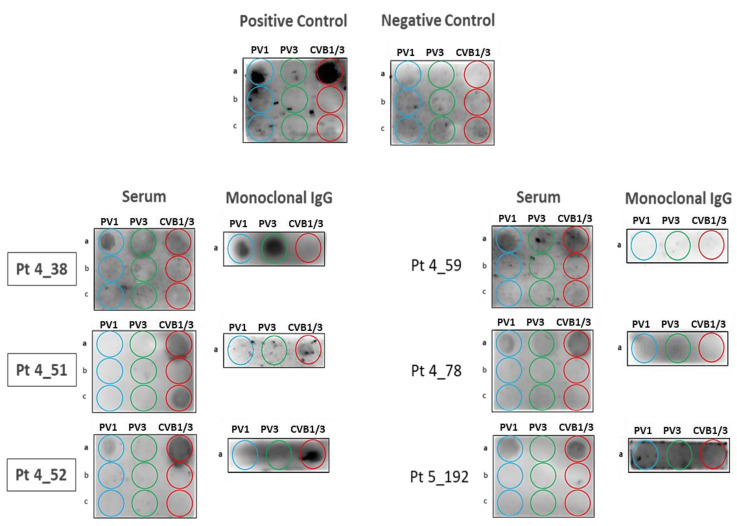
Results of the “PV/CVB” Dot Blotting Assays obtained for the 6 Monoclonal Igs found to be Specific for the PALTAV/AETG Epitopes with the PEPperCHIP*^®^* Infectious Epitope Arrays. Membranes were spotted with the following peptides: (**a**) HP-1a, HP-3a and HC-a, respectively relevant to PV1, PV3 and CVB1/B3; (**b**) irrelevant HP-1b, HP-3b and HC-b peptides; (**c**) irrelevant HP-1c, HP-3c and HC-c peptides (see Supplementary Table S2), then incubated with positive or negative controls or samples of serum from patients. After validation that no signal was obtained with irrelevant peptides for serum samples, purified monoclonal Igs were incubated with relevant peptides only. Immunoblot revelation with secondary antibodies (Materials & Methods). The assay confirmed that the purified monoclonal IgG of MGUS patients 4_38, 4_51 and 4_52 (in bold, left panel) bound specifically to PALTAVETG or PALTAAETG epitopes.

The “PV/CVB” dot blotting assay was first used to confirm the specificity the 6 monoclonal IgGs identified as specific for the PALTAVETG or PALTAAETG peptides using the PEPperCHIP^®^ arrays. For the 6 patients, serum IgGs bound to at least one of the relevant “PV1,” “PV3” or “CVB” peptides (Figure 3). Serum samples did not react with the 6 irrelevant peptides. When purified monoclonal IgGs were analyzed, only 3/6 bound to relevant PV/CVB peptides. One monoclonal IgG gave a strong signal for the “PV3” peptide and a weak signal for the “PV1” peptide (Pt 4_38) and 2 recognized only the “CVB1/3” peptide (Pt 4_51, Pt 4_52). Although repeated several times, the PV/CVB blotting assay was negative for 3 monoclonal IgGs (4_59, 4_78, 5_192). The lack of confirmation by dot blotting assay of the PEPperCHIP^®^ array results for 3 patients may be explained by the difference in quantity of monoclonal Ig used in each assay (10 µg for the dot blotting assay, 50 or 100 μg for PEPperCHIP^®^ arrays). However, for the rest of the study, the monoclonal IgGs of patients 4_59, 4_78 and 5_192 were considered of undetermined specificity.

### 3.4. Screening of MGUS/SM and MM Cohorts for Poliovirus and Coxsackievirus VP1 Recognition by Serum Antibodies and Purified Monoclonal Igs

We first examined the serum of 40 healthy donors (HD) over 60, in order to determine the frequency of anti-PALTAVETG or anti-PALTAAETG antibodies in a healthy, control population. Supplementary Figure S4 shows that 18/40 HD (45.0%) had antibodies that bound to at least one of the 3 relevant peptides. Seventeen HD (42.5%) had antibodies to the “PV1” peptide; 7 had antibodies to the “PV3” peptide and 16 HD (40.0%) had antibodies to the “CVB1/3” peptide.

**Figure 4:**
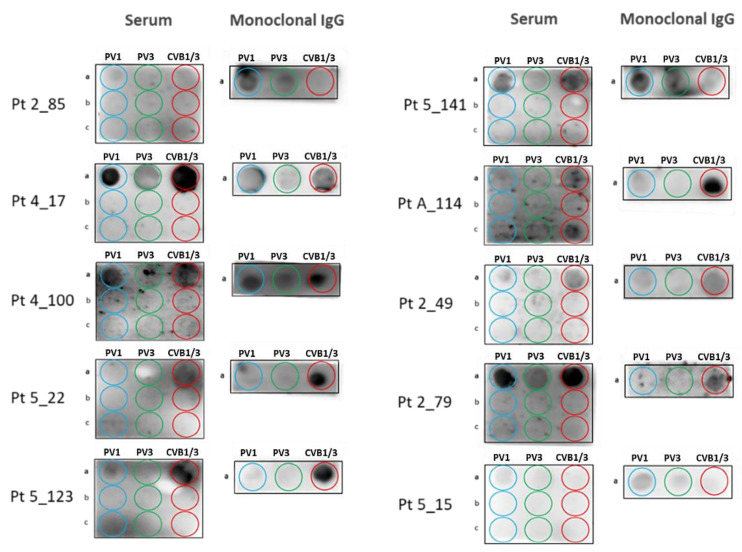
Results of the “PV/CVB” Dot Blotting Assays obtained for the 10 Additional Patients Who Presented a Monoclonal Ig Specific for PV1, PV3 or CVB1/3 Peptides. Membranes were spotted with nine peptides: (**a**) HP-1a, HP-3a and HC-a, respectively relevant to PV1, PV3 and CVB1/B3; (**b**) irrelevant HP-1b, HP-3b and HC-b peptides; (**c**) irrelevant HP-1c, HP-3c and HC-c peptides (see Supplementary Table S2), then incubated with serum (left) or purified monoclonal Ig (right), followed by revelation (see Materials and Methods). After validation that no signal was obtained with irrelevant peptides for serum samples, purified monoclonal Igs were incubated with relevant peptides only. Patients 2_49, 2_79, 5_15 are NDMM; all others are MGUS.

We then examined the serum of patients (Supplementary Figure S5, Figure 4). Samples of serum were available for 76/155 (49.0%) MGUS/SM patients (73 MGUS, 3 SM) and 86/147 (58.5%) MM patients (49 NDMM, 37 RRMM). Only 28/76 (36.8%) MGUS/SM and 16/86 (18.6%) MM patients had serum antibodies to at least one of the “PV1,” “PV3” or “CVB1/3” peptides. In the MM cohort, 17/49 (34.7%) NDMM had antibodies to at least one relevant peptide vs. 4/37 (10.8%) RRMM.

Among the 28 MGUS/SM patients with a positive “PV/CVB” dot blotting assay, 21 (75.0%) had serum antibodies to the “PV1” peptide; 16 (57.1%) had antibodies to the “PV3” peptide; and 19 (67.9%) had antibodies to the “CVB1/3” peptide. Among the 16 MM patients with a positive PV/CVB” dot blotting assay, 13 (81.2%) had serum antibodies to the “PV1” peptide; 9 (56.2%) had antibodies to the “PV3” peptide; and 12 (75.0%) had antibodies to the “CVB1/3” peptide. Thus, the majority of MGUS/SM and MM patients with a positive “PV/CVB” assay had antibodies that bound to the 3 relevant PV/CVB peptides. The “PV/CVB” dot blotting assay was then used to analyze the purified monoclonal Igs of the 28 MGUS and 16 MM patients who presented with serum antibodies against the “PV1”, “PV3” or “CVB1/3” peptides. These studies allowed to identify 10 additional patients (7 MGUS, 3 NDMM) as presenting with a monoclonal Ig that specifically recognized the “PV” PALTAVETG or/and “CVB” PALTAAETG peptides (Figure 4). All 10 patients had a monoclonal IgG. For patients 2_85, 5_141 and 5_15, the monoclonal IgG gave the strongest signal for the “PV1” PALTAVETG peptide. For patients 4_17 and 4_100, signals were similar for the “PV1” and “CVB” peptides; a weak signal was also observed for the “PV3” peptide. For the 5 other patients (5_22, 5_123, A_114, 2_49, 2_79), the monoclonal IgG gave the strongest signal for the “CVB” PALTAAETG peptide. For Pt 2_49, weak signals were noted for PV peptides.

### 3.5. Characteristics of Patients with a PALTAV/AETG-Specific Monoclonal Ig

Thirteen patients (9 MGUS, 1 SM, 3 NDMM) presented with a PALTAV/AETG-specific monoclonal Ig. Analysis of their characteristics, limited due the small size of cohorts and paucity of available clinical information, did not reveal significant differences in biological or clinical presentation for MGUS/SM patients (Table 2). The 3 MM patients with PALTAV/AETG-specific monoclonal IgG were women with stage I MM disease. Of note, 4 MGUS/SM and 1 MM patients with a PALTAV/AETG-specific clonal IgG had non-clonal anti-GlcSph autoantibodies in serum.

### 3.6. Comparison of Monoclonal Gammopathies Linked to the PALTAV/AETG Epitopes vs. Monoclonal Gammopathies Linked to Infectious Pathogens Other Than Enteroviruses or Linked to GlcSph

**Figure 5:**
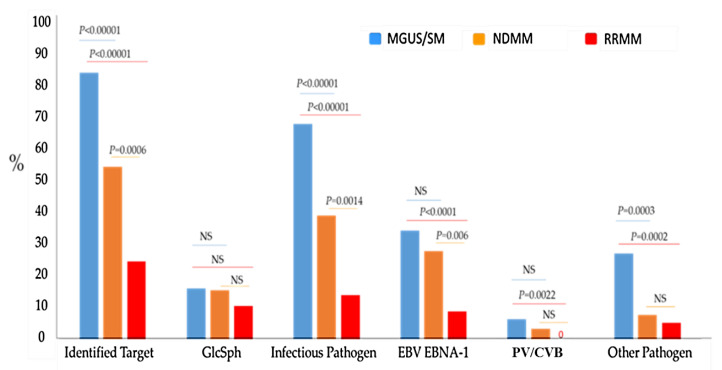
Percentages of Patients with a Monoclonal Ig Specific for GlcSph or an Infectious Pathogen in Cohorts of MGUS/SM, newly diagnosed MM (NDMM) or refractory MM (RRMM). Targets of Monoclonal Igs: GlcSph, glucosylsphingosine; EBNA-1, EBV Nuclear Antigen 1; PV/CVB: PALTAVETG or PALTAAETG sequences of human PV1/3 and CVB1/B3; Other pathogen: HSV-1, CMV or *H. pylori*. The Chi-2 test was used to compare the percentages of patients in the MGUS/SM group and in NDMM and RRMM groups; *p* < 0.05 was considered significant. NS: Not significant.

Since myeloma is always preceded by a MGUS stage, the percentages of MGUS and MM patients with a monoclonal Ig found to be specific for a particular target antigen provide information on whether target antigens influence the risk of progression from MGUS toward MM. Altogether, we were able to determine the target of the patient’s monoclonal Ig for 83.5% MGUS/SM, 54.4% NDMM and 24.6% RRMM (Figure 5). The presence of PALTAV/AETG-specific monoclonal Igs is not rare in MGUS (6.5% cases) and seems less frequent in MM (2.0%; difference not significant, *p* = 0.08, *Chi-2* test). Other infectious targets of monoclonal Igs such as herpesviruses, particularly HSV-1 and CMV, have low frequencies in MM compared to MGUS cohorts (Figure 5) [6,9]. These findings suggest that evolution toward MM may be infrequent for MGUS initiated by chronic stimulation by antigens from HSV-1, CMV and possibly, from PV and CVB. In contrast, GlcSph- and EBV EBNA-1-specific monoclonal Igs were reported at similar frequencies in MGUS and in NDMM: in our cohorts, EBV-initiated disease represented 34.2% of MGUS and 27.8% of NDMM and GlcSph-initiated disease represented 16.1% of MGUS and 15.5% of NDMM (Figure 5). Thus, most EBV EBNA-1- and GlcSph-initiated MGUS appear to eventually progress toward MM [6,9,10]. Intriguingly, EBV EBNA-1 was a rare target of monoclonal Igs in the RRMM cohort (8.8% for RRMM vs. 27.8% for NDMM, *p* = 0.006, *Chi-2* test) (Figure 5).

## 4. Discussion

Enteroviruses are single-stranded RNA viruses, named by their transmission route through the intestine. They are classified into four groups: *Enterovirus A, Enterovirus B* (which includes coxsackieviruses CVB1–CVB6), *Enterovirus C* (which includes polioviruses PV1–PV3) and *Enterovirus D*; the genotyping of enteroviruses isolated recently is based on the VP1 capsid region [18]. Enterovirus infection may result in a variety of symptoms, ranging from mild respiratory illness (common cold) to meningitis and acute flaccid paralysis and myelitis [19]. In particular, poliomyelitis (“polio”) may be caused by any of the 3 polioviruses (PV1, PV2 or PV3), which all produce the same symptoms. Poliovirus type 1 (PV1) is the most frequent and the most associated with paralysis. In the 1950s, two types of anti-polio vaccines (attenuated, inactivated) were developed; following anti-polio vaccination, 90% of individuals produced protective antibodies to the three polioviruses [20,21,22,23]. Recent studies of the general population of Europe and the USA typically report seroprevalences ≥90% for PV1 and PV2 and ≥80% for PV3 in adults (vaccinated or not) and seroprevalences ≥94% for the three polioviruses in young children [24,25,26]. Regarding coxsackieviruses of B type (CVB), seropositivity for at least one CVB was detected in 69% individuals in a recent study, with some variability: 33.3% for CVB3, 26.2% for CVB5, 12.7% for CVB1, 11% for CVB2 [27].

Our own study focused on the PALTAVETG epitope of the VP1 protein of PV1 and PV3 and the VP1 PALTAAETG epitope of CVB1 and CVB3. However, the PALTAVETG and PALTAAETG sequences are shared by many more picornavirus. The PALTAVETG sequence is found in human enterovirus C96, coxsackieviruses A13 and A24 and echovirus E11, as well as in rat and rabbit picornavirus. The PALTAAETG epitope is found in human CVB1, CVB3, CVB5 and CVB6 and in echoviruses E6, E9, E16, E17, E20, E31, E87 and B85, as well as in bat and porcine sapelovirus (which cause neurologic disease in animals). In our cohort of healthy individuals over 60 years of age, 45% presented with serum antibodies directed against PALTAVETG and PALTAAETG epitopes. Several reasons may explain the low prevalence. First, we studied only 2 epitopes; second, seropositivity may be underestimated because we assigned a negative score to individuals with weak reactivity in the “PV/CVB” dot blotting assay. Of note, 36.8% MGUS/SM patients and 34.7% NDMM patients carried serum antibodies to the PALTAVETG/PALTAAETG epitopes, proportions similar to the one observed for healthy individuals. In contrast, the % RRMM patients with anti-PALTAVETG/ PALTAAETG antibodies in serum was lower (10.8%), likely reflecting the reduced production of polyclonal antibodies typical of advanced stages of MM. Like healthy donors, most of the MGUS and MM patients with a positive serology had antibodies against both the PALTAVETG (PV) and PALTAAETG (CVB) epitopes.

Chronic antigenic stimulation is increasingly recognized as a pathogenic mechanism in a significant fraction, possibly half, of B-lineage malignancies, including myeloma. Our study is the first to show that monoclonal Igs may target the VP1 PALTAVETG and PALTAAETG epitopes of PV1/3 and CVB1/B3 in MGUS (6.5% cases) and to a lesser degree, in MM. These data suggest that abnormal B-cell responses against the PALTAV/AETG epitopes may initiate subsets of MGUS. We were not able to determine whether a specific virus triggers the monoclonal IgG response, since most of the monoclonal IgGs we studied bound to both the PALTAVETG and PALTAAETG epitopes. Interestingly, PALTAV/AETG-initiated MGUS seem to unfrequently progress toward MM: in our cohorts, only 2.0% MM presented with a monoclonal Ig specific for the PALTAV/AETG epitopes. Based upon our previous studies, evolution toward MM also appears to be infrequent for MGUS initiated by chronic antigenic stimulation by viruses such as HSV-1 and CMV. In contrast, EBV- and GlcSph-initiated MGUS seem to eventually progress toward MM [6,9,10]. In fact, many studies have established that significant differences exist among MGUS and SM patients both in genetics and in early exposure to viruses, especially EBV, with consequences on immune responses, MGUS clinical presentation and risk of transformation into MM [28,29,30,31,32]. In this regard, the determination of the target of the monoclonal Ig in large cohorts of patients, which is feasible for > 80% MGUS/SM patients and > 50% NDMM patients (Figure 5), should contribute to better define the prognosis of MGUS patients and subsequently, influence therapeutic choices to prevent MM.

We propose that these successful methods to identify targets of monoclonal Igs in MGUS and SM can lead to novel treatments based upon reducing target antigen burden. This new approach has been proven successful in the context of HCV- and GlcSph-initiated MGUS and also in MM cases. For patients with HCV-associated MGUS or MM, antiviral treatment led to reduction or the disappearance of both monoclonal Ig and plasmacytic clone [12,13]. Similarly, in two patients with GlcSph-associated MGUS or MM, treatments designed to reduce GlcSph levels led to the disappearance of the clonal Ig and plasma cells [11]. At present, we lack strategies to reduce antigen exposure in patients whose monoclonal Ig reacts with PV or CVB.

It is notable that the patient’s monoclonal Ig is not yet identified for 75% of RRMM patients. Since clonal plasma cells acquire numerous genetic alterations during MM disease progression, one hypothesis to explain this may be that alterations of Ig-encoding genes accumulate over time and result in decreased or loss of binding affinity of the monoclonal Ig for its initial target antigen.

In conclusion, the PALTAVETG and PALTAAETG sequences of VP1 coat proteins of human PV1/PV3 and CVB1/CVB3 are the targets of subsets of monoclonal Igs and likely initiate ≥ 6% of MGUS cases. PALTAV/AETG-initiated MGUS seem to rarely evolve toward myeloma.

## Figures and Tables

**Table 1:** PEPperCHIP*^®^* Infectious Epitope MicroArray Results of monoclonal gammopathy of undetermined significance (MGUS)/smoldering myeloma (SM) Monoclonal IgGs.

Patient	Pathogen	Protein	Sequence
4_07 ^1^	EBV	EBNA-1	PPRRPPPGRRPFFHPVG
2_91	HSV-2	Envelope glycoprotein G	PEEFEGAGDGEPPED
4_09	HSV-1	Envelope glycoprotein G	TPPMPSIGLEEEE
4_11	EBV	EBNA-1	PPGRRPFFHPVGEADYF
4_12	HSV-1	Envelope glycoprotein G	EGAGDGEH + LEGGD
4_19	EBV	EBNA-1	GRRPFFHPVGEADYFEY
4_38	*Enterovirus C*	VP1 coat protein	EIPALTAVETGATNP
4_51	CVB1	VP1 coat protein	IPALTAAETGHTSQV
4_52	*Enterovirus C*	VP1 coat protein	EIPALTAVETGATNP
4_59	CVB1	VP1 coat protein	IPALTAAETGHTSQV
4_78	CVB3	VP1 coat protein	PALTAAETG
4_89 ^2^	HSV-1	Envelope glycoprotein G	MPSIGLEEEEEEE
4_98	HSV-1	Envelope glycoprotein G	LPQSPGPAFPLAE
5_192	CVB3	VP1 coat protein	PALTAAETG

^1^ Positive control: Patient with an EBV EBNA-1 specific monoclonal IgG, as assessed with the MIAA assay; CVB: coxsackievirus B; ^2^ SM patient; all other patients in the table are MGUS patients; VP: viral protein.

**Table 2:** Characteristics of Patients with a PALTAV/AETG-Specific Monoclonal IgG.

Patient Parameters	MGUS/SM	MM
**Men/Women** (% men) **Age** (years) Median (Min-Max) **Amount of Mc Ig** (g/L) Median (Min-Max) **BM plasma cells** (%) Median (Min-Max) **β_2_-microglobulin** (mg/L) Values **Bone lesions** Number with lesions (%) **ISS Stage** Stage I (%) **DSS Stage** Stage I (%) **Anti-GlcSph antibodies** Positive (%)	4/4 (50%) n = 9 68.0 (38.2–84.9) n = 10 17.1 (8.0–37.4) n = 4 3.5 (1.0–5.0) n = 1 2.1 NA NA NA NA NA NA 4/10 (40.0%)	0/3 (0%) n = 3 70.0 (65–71) n = 3 18.0 (12–25.2) n = 2 28.1 (26.5–29.6) n = 2 2.1-3.2 n = 3 0 (0%) n = 2 2 (100%) n = 3 3 (100%) 1/3 (33.3%)

Mc Ig: purified monoclonal Ig; BM: bone marrow; NA: not applicable. Because complete information was not available for all patients, the number (n) of patients with data varies depending on the parameter.

## Data Availability

The data presented in this study are available on request from the corresponding author.

## Supplementary Materials

The following are available online at https://www.mdpi.com/2073-4409/10/2/438/s1, Table S1: Characteristics of the MGUS/SM and MM cohorts, Table S2: Detail of the peptides derived from Human Enteroviruses, Figure S1: Results of the Glucosylsphingosine (GlcSph) Assay of the 6 RRMM patients with a GlcSph-reactive Monoclonal IgG, Figure S2: Results of PEPperCHIP*^®^* Infectious Epitope arrays obtained for monoclonal IgGs specific for EBV EBNA-1, Figure S3: Results of PEPperCHIP*^®^* Infectious Epitope arrays obtained for monoclonal IgGs specific for HSV-1/2 envelope glycoprotein G, Figure S4: Results of “PV/CVB” dot blotting assays obtained for healthy donors, Figure S5: Positive “PV/CVB” dot blotting assays from MGUS/SM and MM patients.
