# Supplementary material for: Comparison of Monoclonal Gammopathies Linked to Poliovirus or Coxsackievirus vs. Other Infectious Pathogens
Source: Cells. 2021 Feb 19;10(2):438. doi: 10.3390/cells10020438 (PMC7922508; doi:10.3390/cells10020438)
Supplement: Supplementary file 1 [file cells-10-00438-s001.pdf]

cells

Supplementary Materials

Supplementary Table S1. Characteristics of MGUS/SM and MM Cohorts.

**Characteristics of Patients**	**MGUS/SM (Nbr = 155)**	**NDMM (Nbr = 90)**	**RRMM (Nbr = 57)**
**Sex**			
Nbr	141	90	40
M/F (male %)	80/61 (56.7%)	50/40 (55.5%)	12/28 (30.0%)
**Age** (years)			
Nbr	141	90	39
Median (Min-Max)	68.7 (31-95)	67.0 (42-92)	61.0 (46-83)
**Amount of Mc Ig** (g/L)			
Nbr	152	90	29
Median (Min-Max)	16.0 (4.0-39.8)	23.0 (4.0-68.0)	34.8 (10.3-88.5)
**β_2-microglobulin** (mg/L)			
Nbr	46	86	ND
Median (Min-Max)	2.4 (1.1-10.1)	3.1 (1.3-14.0)	ND
**Bone lesions**			
Nbr	68	86	ND
Positive (%)	5 (7.4%)	59 (68.6%)	ND
**ISS Stage**			
Nbr	NA	50	ND
Stage III (%)	NA	14 (28.0%)	ND
**DSS Stage**			
Nbr	NA	73	ND
Stage III (%)	NA	32 (43.8%)	ND
**Target of Mc Ig** (Nbr (%))			
Identified Target	120 (77.4%)	46 (51.1%)	14 (24.6%)
- GlcSph	25 (16.1%)	14 (15.5%)	6 (10.6%)
- MIAA Infectious Pathogen	95 (61.3%)	32 (35.6%) [a]	8 (14.0%) [b,c]
EBV EBNA-1	53 (34.2%)	25 (27.8%)	5 (8.8%)
Other infectious epitopes	42 (27.1%)	7 (7.8%)	3 (5.2%)
Unknown Target	35 (22.6%)	44 (48.9%)[d]	43 75.4%)[e,f]

Nbr: number of patients; Mc Ig = purified monoclonal Ig; NA: not applicable; ND: no data. Because complete information was not available for all patients, the number of patients with data varies depending on the parameter. Notably, for most MM patients cytogenetics data were not available. (a) *P*=0.0001 *vs* MGUS/SM, (b) *P*<0.00001 *vs* MGUS/SM, (c) *P*=0.0043 *vs* NDMM, *Chi-2* square test; (d) *P*<0.0001 *vs* MGUS/SM, (e) *P*<0.0001 *vs* MGUS/SM, (f) *P*=0.0014 *vs* NDMM, *Chi-2* square test.

Supplementary Table S2. Peptides Derived from Human Enteroviruses.

Peptide Name	Amino-Acid Sequence	Targeted Human Enteroviruses
**HP-1a**	THSKE**IPALTAVE**TGATN	**Human poliovirus 1 (PV1), Mahoney Strain**
HP-1b	THSKE**I**E**A**EP**A**E**E**TGATN	HP-1a-derived irrelevant sequence (b)
HP-1c	THSKE**IP**F**L**F**AVE**TGATN	HP-1a-derived irrelevant sequence (c)
**HP-3a**	AHSKE**VPALTAVE**TGATN	**Human poliovirus 3 (PV3), Sabin Strain**
HP-3b	AHSKE**V**E**A**EP**A**E**E**TGATN	HP-3a-derived irrelevant sequence (b)
HP-3c	AHSKE**VP**F**L**F**AVE**TGATN	HP-3a-derived irrelevant sequence (c)
**HC-a**	TNSES**IPALTAA**ETGHTS	**Human coxsackieviruses B1/B3 (CVB1/3)**
HC-b	TNSES**I**E**A**EP**AA**ETGHTS	HCa-derived irrelevant sequence (b)
HC-c	TNSES**IP**F**L**F**AA**ETGHTS	HCa-derived irrelevant sequence (c)

HP-1a, HP-3a and HC-a peptides are relevant peptides; all other peptides are control (irrelevant) peptides.

Supplementary Figure S1: Results of the Glucosylsphingosine (GlcSph) Assay of the 6 RRMM patients with a GlcSph-reactive Monoclonal IgG.

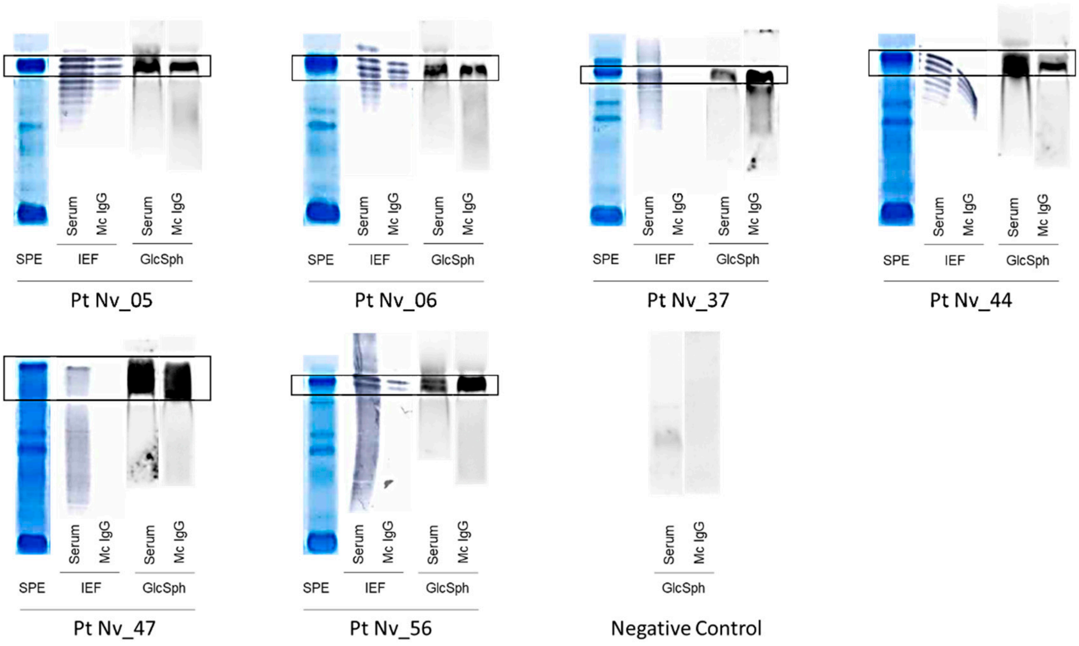

Measurement of Ig concentration, separation of monoclonal Igs from other Igs, and verification of purity were performed as described in Materials & Methods. Purification started with the separation of serum proteins with high resolution agarose gel electrophoresis (SAS-MX high resolution, Helena Biosciences, Gateshead, UK). Then, the monoclonal Ig was cut from the gel and eluted in PBS. The purity of the monoclonal Ig preparation was verified by isoelectric focusing (IEF) on an agarose gel (pH 3–10) followed by blotting and immuno-revelation by an anti-human IgGγ chain antibody labeled with peroxidase. For RRMM patients Nv_37 and Nv_47, images of the IEF are missing. For GlcSph assays, polyvinylidene fluoride (PVDF) membranes were incubated for 90 min in 100 µg/mL of GlcSph in 0.1 M sodium bicarbonate, rinsed in PBS and 0.1% Tween 20 detergent, and then blocked with 5% BSA in PBS and 0.1% Tween 20. Samples of serum and purified monoclonal Ig were submitted to agarose gel electrophoresis, and then the gels were blotted onto GlcSph-saturated membranes by diffusion blotting during 12 min. After blocking with 2.5% BSA in PBS, membranes are incubated with anti-human IgG HRP-conjugated secondary antibody, washed, and revealed by chemiluminescence. Signals corresponding to the patient's monoclonal IgG are encircled in black. The negative control shows a patient with no GlcSph-reactive Ig in serum. SPE = Serum Protein Electrophoresis; Mc Ig = Purified Monoclonal Ig.

Supplementary Figure S2. Results of PEPperCHIP® Infectious Epitope MicroArrays obtained for monoclonal IgGs specific for EBV EBNA-1.

Patient 4_07

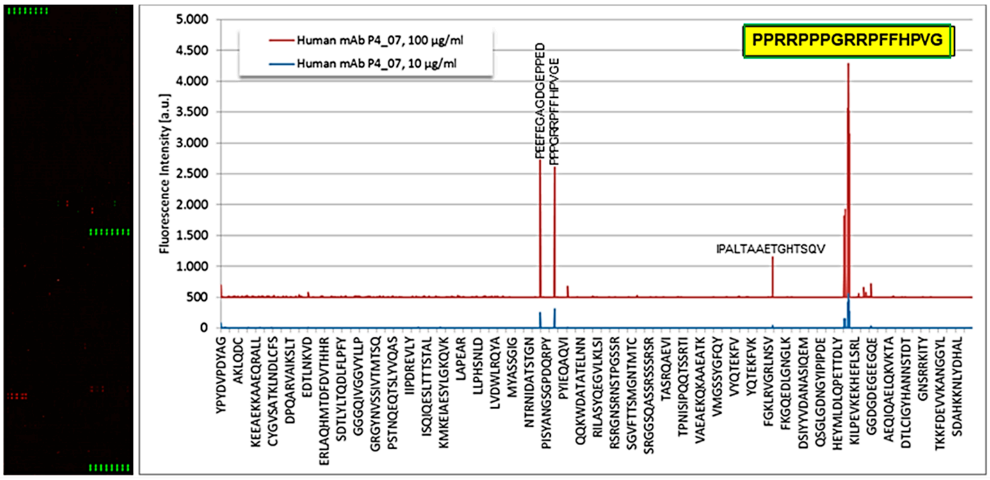

Patient 4_11

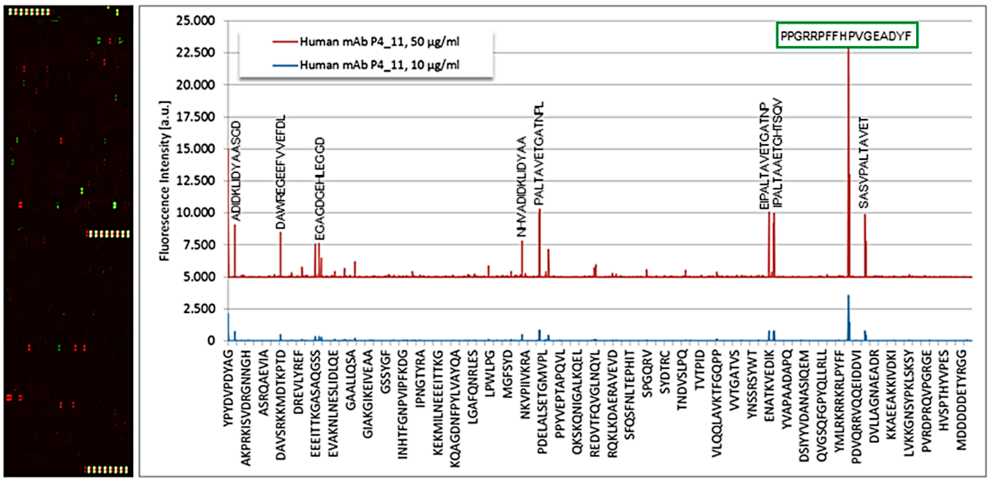

Patient 4_19

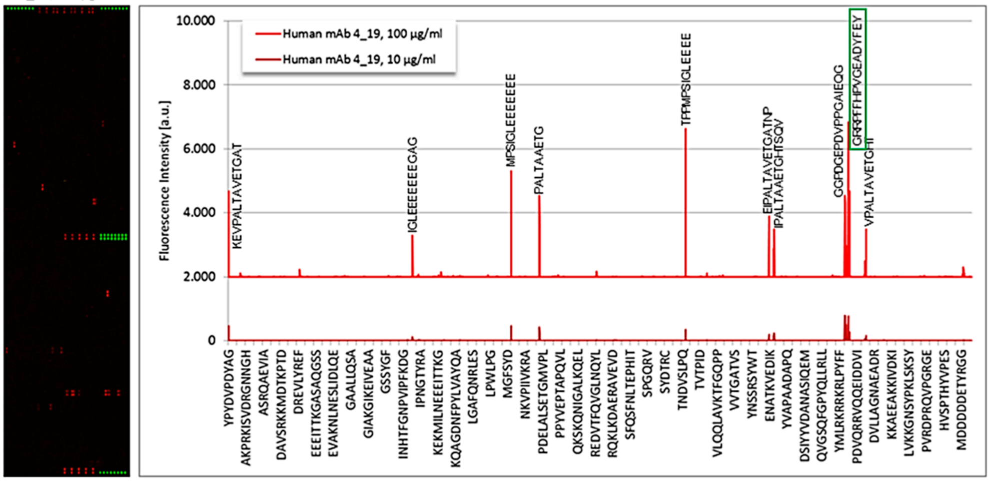

Microarrays were incubated with the patient monoclonal IgG (50 or 100 µg/mL), followed by staining with secondary and control antibodies and read at scanning intensities of 7/7 (red/green) as described in Methods. Antibody response against peptides is annotated next to the corresponding signal in the intensity plot (left panel). Well-defined staining of HA control peptides appear in green, the PV controls appear in red.

Supplementary Figure S3. Results of PEPperCHIP® Infectious Epitope MicroArrays obtained for Monoclonal IgGs specific for HSV-1/2 Envelope Glycoprotein G.

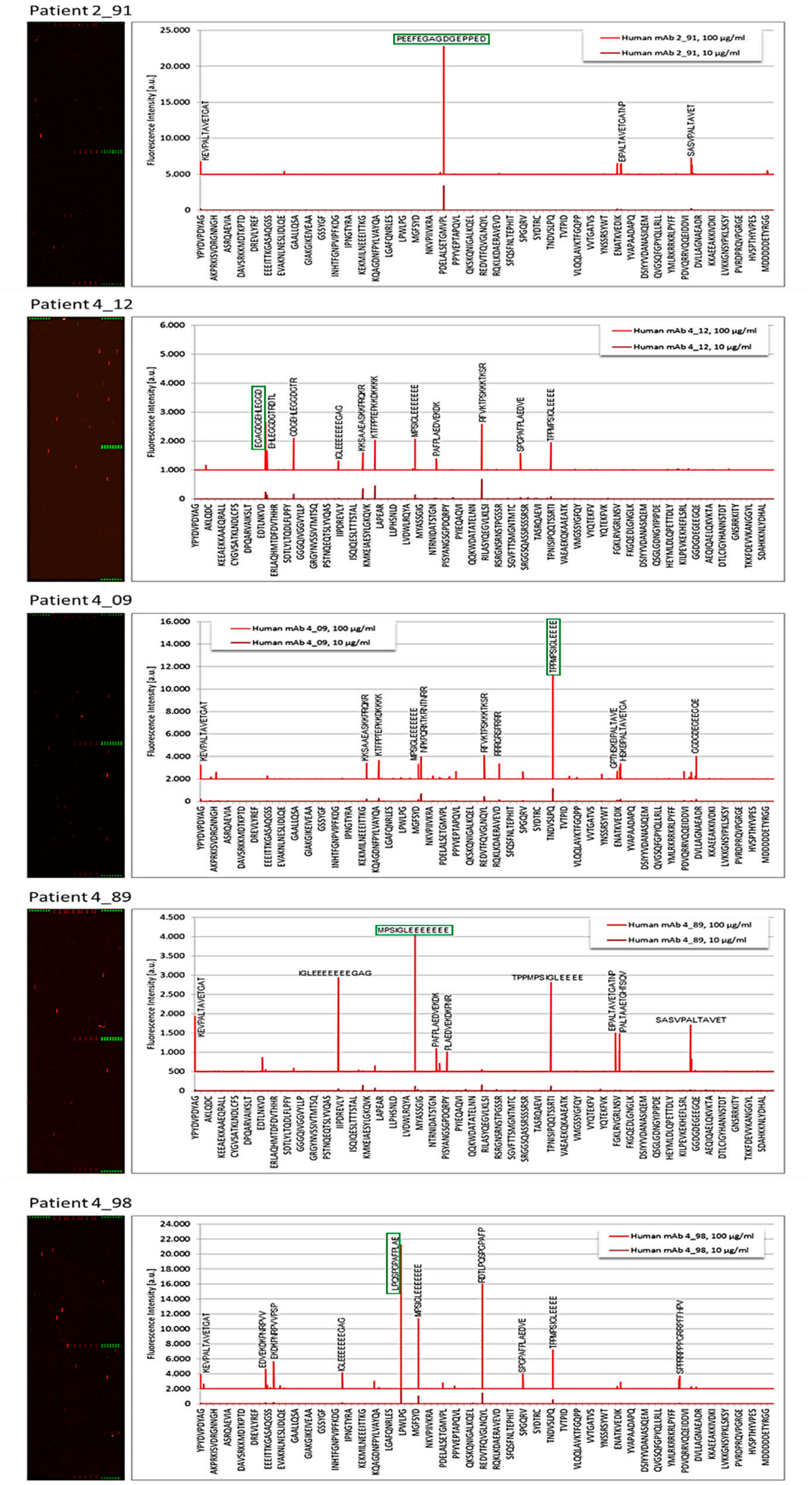

Microarrays were incubated with the patient monoclonal IgG (100 µg/mL), followed by staining with secondary and control antibodies and read at scanning intensities of 7/7 (red/green). Antibody response against peptides is annotated next to corresponding signals in the intensity plot (left panel). Well-defined staining of HA control peptides appear in green, the PV controls appear in red.

Supplementary Figure S4. Results of "PV/CVB" Dot Blotting Assays obtained for Healthy Donors.

Part A

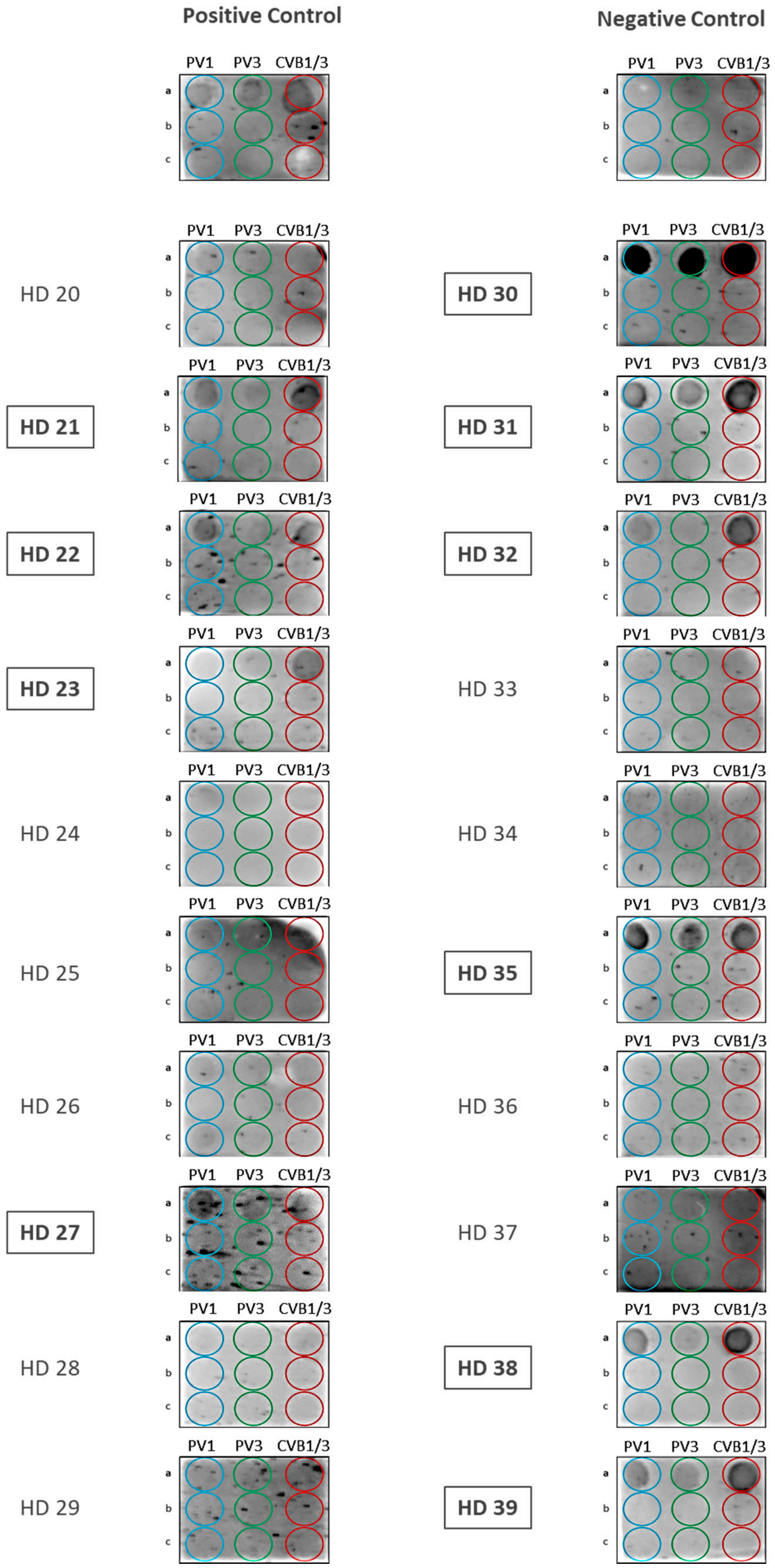

Part B

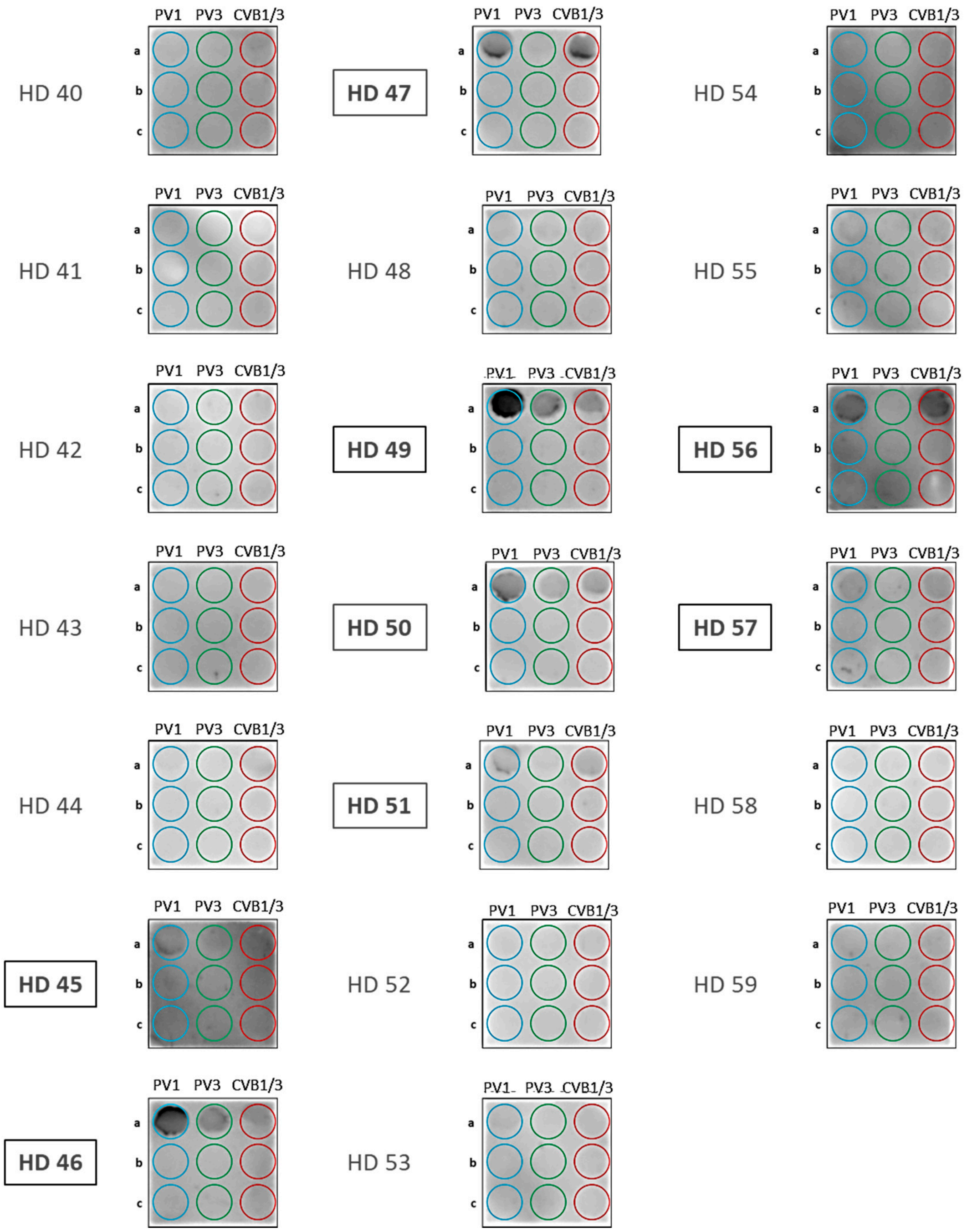

Membranes were spotted with nine peptides, as indicated: (a) HP-1a, HP-3a and HC-a, respectively relevant to PV1, PV3 and CVB1/B3; (b) irrelevant HP-1b, HP-3b and HC-b peptides ; (c) irrelevant HP-1c, HP-3c and HC-c peptides (see Supplementary Table S2), then incubated with samples of serum from 40 healthy donors (HD), followed by revelation with secondary antibodies, as described in Materials and Methods. Parts A and B differ only by the size of membranes, smaller in Part B. The 18 healthy individuals with antibodies in serum that bound to at least one of the relevant HP-1a, HP-3a, or HC-a peptides, are shown in a black frame.

Supplementary Figure S5. Positive "PV/CVB" Dot Blotting Assays from MGUS and MM Patients.

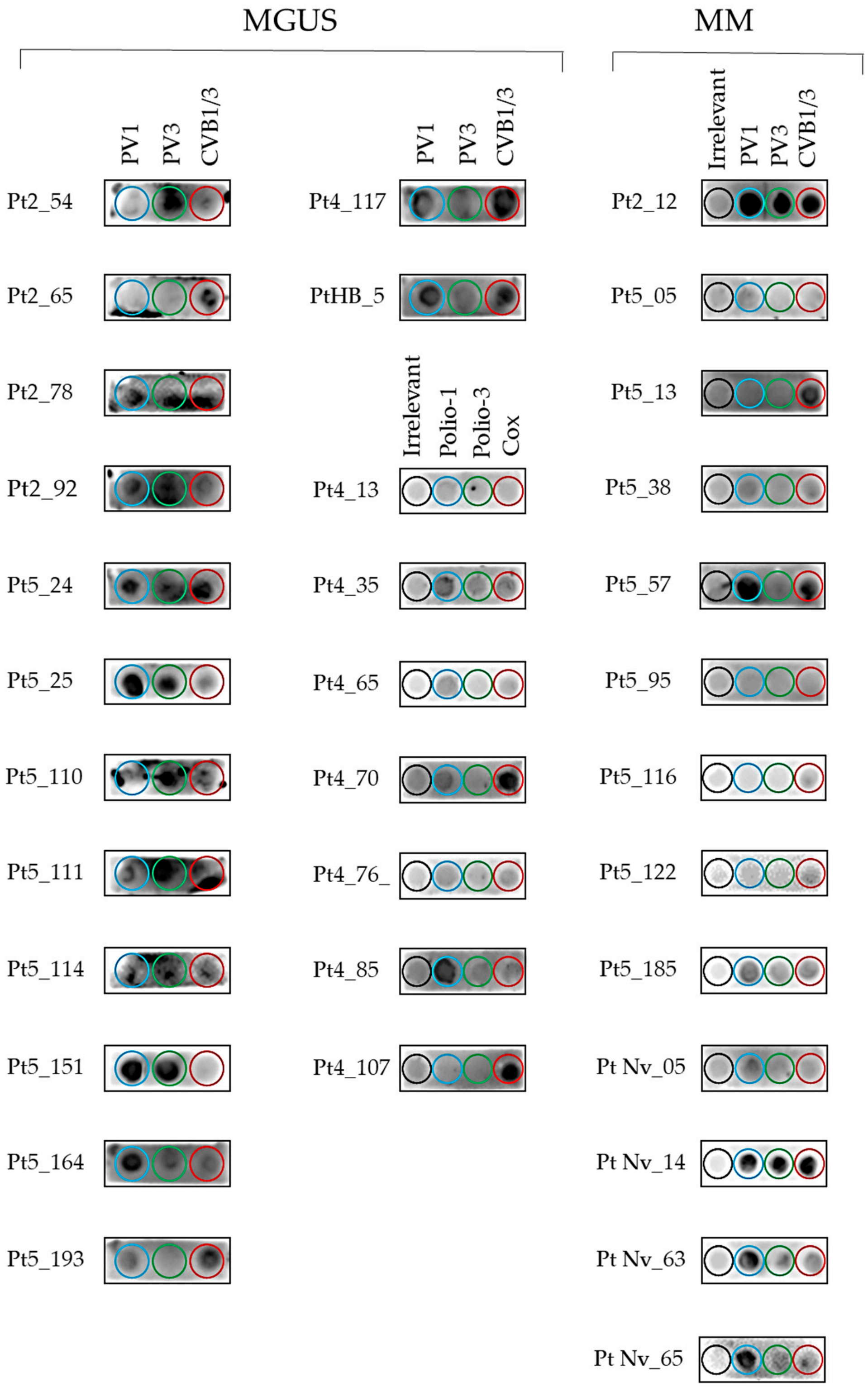

Membranes were spotted either with three peptides, as indicated: HP-1a, HP-3a and HC-a, respectively relevant to PV1, PV3 and CVB1/B3; or with a mix of six irrelevant peptides (HP-1b, HP-3b, HC-b, HP-1c, HP-3c, HC-c) used as a negative control, along with the three relevant HP-1a (PV1), HP-3a (PV3) and HC-a (CVB1/3) peptides (see Supplementary Table S2 for peptide detail). Spotted membranes were then incubated with samples of serum from patients, followed by revelation with secondary antibodies, as described in Materials and Methods. Patients 2_12 to 5_185 are NDMM patients; patients Nv_05, Nv_14, Nv_63 and Nv_65 are RRMM patients.
